# The double-edged sword - prosthetic joint infection following BCG treatment for bladder cancer: a case report

**DOI:** 10.1186/s12879-019-3951-1

**Published:** 2019-04-18

**Authors:** Minh-Vu Hoang Nguyen, Mauro M. Giordani, George R. Thompson

**Affiliations:** 1 University of California Davis Medical Center, Sacramento, CA USA; 2 Department of Orthopedics, University of California Davis Medical Center, Sacramento, CA USA; 3 Department of Medical Microbiology and Immunology, University of California – Davis, University of California Davis Medical Center, Sacramento, CA USA; 4 Department of Internal Medicine, Division of Infectious Diseases, University of California Davis Medical Center, Sacramento, CA 95817 USA

**Keywords:** Prosthetic joint infection, BCG, *Mycobacterium bovis*

## Abstract

**Background:**

Prosthetic joint infections remain a significant cause of morbidity and are frustrating for patients and physicians alike. Unusual causes of infection may be seen in selected circumstances and a high index of suspicion and a careful history are required to ensure an accurate and timely diagnosis can be made.

**Case presentation:**

We present a case of *Mycobacterium bovis* prosthetic joint infection secondary to intravesicular Bacillus Calmette-Guérin (BCG) treatment for prior bladder cancer definitively identified by spoligotyping. A favorable clinical outcome was observed following surgical intervention and a 12-month course of anti-mycobacterial therapy.

**Conclusions:**

BCG therapy, a live attenuated strain of *M. bovis,* has become the mainstay of adjunctive therapy for bladder cancer and infectious complications, including those affecting the musculoskeletal system, may be seen years after initial therapy. An awareness of this complication and appropriate discussions with the institution’s microbiology laboratory may allow for an accurate and timely identification.

## Background

Prosthetic joint infections remain a significant problem and affect 1–2% of all joint replacement surgeries [1]. The majority of these infections are caused by bacterial pathogens such as *Staphylococcus aureus*, gram-negative bacilli, or from mixed infections [2]. The diagnosis of less-common causes of infection requires a careful history and thoughtful discussions with the microbiology laboratory to ensure other potential etiologic agents can be sought and thereafter definitively identified.

### Case presentation

A 90-year-old Hispanic male dairy farmer with a complex medical history notable for a left total hip arthroplasty (THA), bladder carcinoma in situ status-post intravesicular Bacillus Calmette-Guérin (BCG) (a live attenuated strain of *Mycobacterium bovis*) treatment, and chronic kidney disease who presented with subacute worsening pain of his left thigh. He had a THA placed thirty-one years previously. He had papillary bladder tumor status-post fulguration five years prior to admission with subsequent recurrence of bladder carcinoma in situ diagnosed a year later; he underwent six initial and six maintenance instillations of BCG treatment with remission of his bladder cancer.

**Fig. 1:**
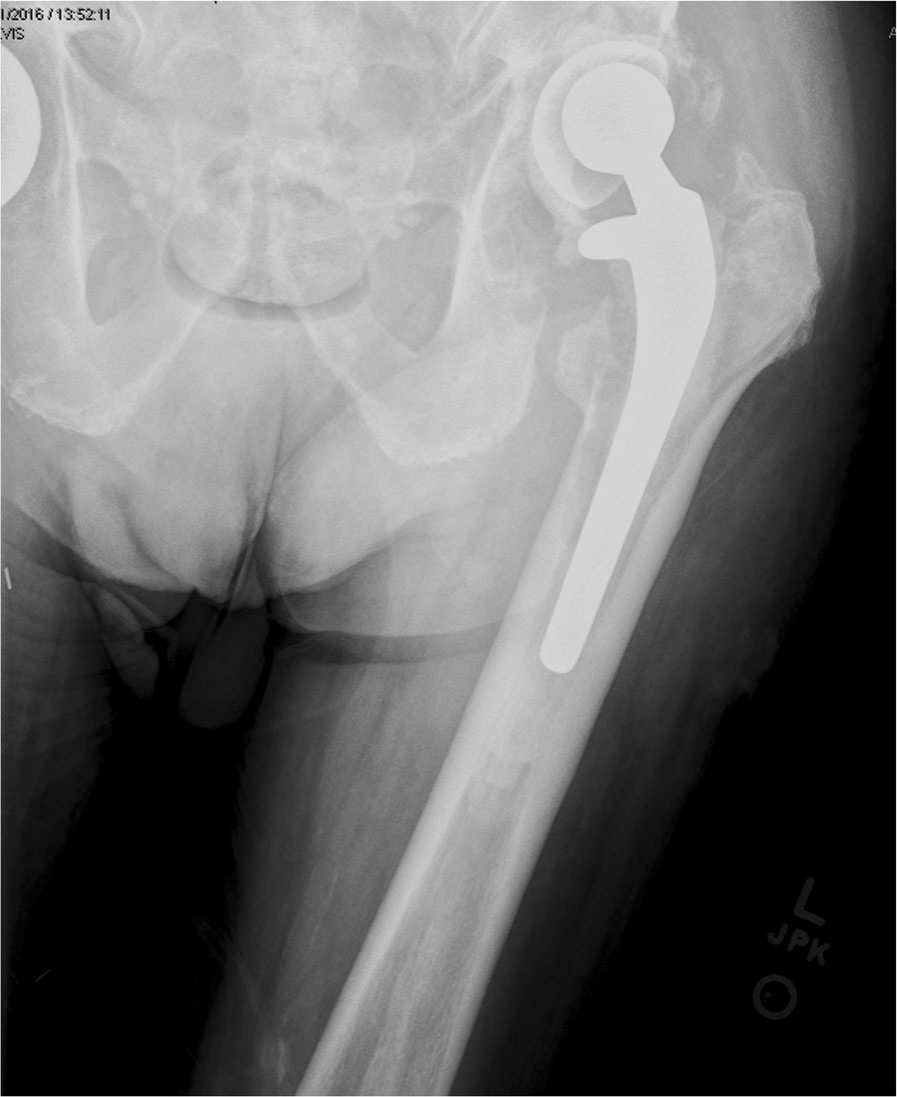
Peri-prosthetic lucency consistent with long-standing infection

Four years after BCG therapy, the patient developed new-onset drainage from the left lateral thigh. This was followed by swelling of his entire left thigh with increasing purulent discharge and pain with movement. He subsequently experienced chills, rigors, and a fever of 101 °F the morning prior to admission. On presentation he was afebrile with normal vital signs. His exam was significant for an open wound on the lateral left thigh with purulent drainage and surrounding erythema. Pain was noted adduction of the left hip. Initial laboratory tests were notable for a white blood cell count of 10,200 cells/mm^3^, a C-reactive protein of 9.7 mg/dL, and sedimentation rate of 71 mm/hr. Radiograph of the left hip showed “extensive lucencies” around the left THA (Fig. 1).

The patient underwent incision and drainage with an antibiotic spacer placed following admission. Wound, hip fluid, and abscess cultures obtained during irrigation and debridement were negative for bacterial pathogens, however given his history of prior BCG therapy the microbiology laboratory was asked to additionally perform mycobacterial cultures and these grew acid-fast bacilli (AFB) concerning for *Mycobacterium tuberculosis* complex. After a brief course of clindamycin, ceftriaxone, vancomycin, and metronidazole, he was started on isoniazid 300 mg PO daily, rifampin 600 mg PO daily, pyrazinamide 1500 mg daily, ethambutol 1200 mg daily, and Vitamin B6 50 mg PO daily. He was placed in temporary airborne isolation while he had three sputa assessed for active pulmonary tuberculosis, which were negative for *Mycobacterium* spp. Nucleic Acid Amplification Testing (Cepheid, Sunnyvale, California) detected *Mtb* complex in his hip fluid culture.

Susceptibility testing for *Mtb* complex showed monoresistance to pyrazinamide, which was suggestive of *M. bovis*. As the patient had prior exposure to livestock and BCG therapy the isolate was further evaluated by spacer oligonucleotide typing (spoligotyping) and found by the California State Department of Health to be identical to that of the vaccine strain *M. bovis* BCG used previously in the treatment of his bladder cancer. The patient completed a 12 month course of rifampin, isoniazid and ethambutol without event and free of symptomatic, or radiographic recurrence.

## Discussion and conclusions

Our case illustrates an *M. bovis* prosthetic joint infection as a complication of intravesicular BCG therapy. *M. bovis* is a zoonotic mycobacteria related to *Mtb* known to cause tuberculosis in cattle, also known as bovine tuberculosis, and other animals. It is recently better known for its employment in modern medicine as the primary ingredient in the BCG vaccine that protects humans from *Mtb* infection and the intravesicular BCG therapy for bladder carcinoma. Despite our ability to harness this organism for potential therapeutic benefit, [3] it still retains its ability to cause human disease.

*M. bovis* infects humans by either transmission from an infected animal or *M. bovis*-derived therapeutic intervention (BCG). Direct exposure to *M. bovis*-infected animals can lead to tuberculosis in humans; immunocompromised individuals are the most susceptible, notably those with human immunodeficiency virus co-infection [4]. Other risk factors pertinent to our case are Hispanic descent and other immunosuppressed states including chronic kidney disease [5]. Among those who are exposed to cattle a common route of transmission is ingestion of unpasteurized milk from an infected cow, but because of the advent of farming infection control and milk pasteurization, this has become less common [6]. Our patient was infected by his BCG therapy for his bladder carcinoma, which was confirmed by spoligotyping to differentiate from any potential *M. bovis* exposure he may have previously encountered as a dairy farmer.

A wide range of BCG infections as complications from prior therapy for bladder carcinoma have been described [7–11]. Prosthetic joint infections are exceedingly rare, with few cases previously presented and none confirmed by spoligotyping/PCR as BCG [12–20]. The nine cases reviewed consist of middle-to-elderly age patients, eight of which were men, without specified ethnicity who had joint arthroplasties and bladder carcinoma needing instillations of BCG therapy. They generally present months to years after their last BCG instillation with worsening localized pain at the site of their arthroplasties and evidence of local bone lesions on radiograph [12, 14–20]. Only one case had a co-morbid immunosuppressive diagnosis (diabetes) [14], only one had systemic B-symptoms [18], and five had elevated serum measurements of acute phase reactants [13, 15–18]. All cases had microbiological confirmation of *M. bovis* and received appropriate anti-tuberculous treatment in addition to surgical source control. Our case is different than the prior cases as he is the first to have PCR confirmed BCG infection rather than probable infection with BCG (the presence of *M. bovis* in a patient with prior BCG).

Identifying an *M. bovis* infection is both clinically, radiographically, and microbiologically challenging because it is part of the *Mtb* complex with *Mycobacterium tuberculosis*, *Mycobacterium africanum,* and *Mycobacterium microti* [21]*.* Clinicians diagnose *M. bovis* in a similar approach to an *Mtb* infection. Radiographic findings can be non-specific and mimic malignancy, or other infectious causes necessitating a tissue diagnosis for confirmation [22]. initially with microscopy with AFB staining followed by culture and nucleic acid testing of pertinent specimens. Because *M. bovis* is innately resistant to pyrazinamide, it is often suggested based on monoresistance to pyrazinamide on susceptibility testing*;* more advanced molecular genetic testing is required for definitive diagnosis [23]. For this case, spoligotyping was employed to identify and confirm the etiology as BCG [24]. In addition to surgical source control needed for a prosthetic joint infection, treatment for BCG and other mycobacterial infections consists of rifampin, isoniazid, and ethambutol for 12 months [25, 26].

In summary, our case is an elderly man with confirmed BCG prosthetic joint infection from his prior intravesicular BCG therapy. Such complications are exceedingly rare and reinforce the need for a thorough history and a high index of suspicion to make an accurate diagnosis. Clinicians should suspect this infection in elderly or immunocompromised patients with an infected prosthesis and a history of intravesicular BCG therapy. If a mycobacterial culture grows *Mtb* complex with monoresistance to pyrazinamide, clinicians can consider spoligotyping to definitively diagnose BCG and trace the strain to its source.

Prosthetic joint infection from the BCG strain of *M. bovis* is a rare infectious complication of prior BCG therapy and requires a high-index of suspicion. A thorough history and working knowledge of unusual causes of prosthetic joint infection allowed for a rapid diagnosis and initiation of therapy.

## Abbreviations

AFB: Acid-fast bacilli
BCG: Bacillus Calmette-Guérin
PCR: Polymerase chain reaction
THA: Total hip arthroplasty
